# An Unusual Case of Combined Thrombosis and Amegakaryocytopenia Resembling Thrombosis With Thrombocytopenia Syndrome Following COVID-19 Infection in an Unvaccinated Patient

**DOI:** 10.7759/cureus.35530

**Published:** 2023-02-27

**Authors:** Mariam Hassan, Swathi Prakash, Jose Rayas, Jared J Bies, Sukhila Reddy, Sahithi Nadella, Sara Alhariri, Yousf Radwan, Nawar Hakim, Javier Corral

**Affiliations:** 1 Internal Medicine, Texas Tech University Health Sciences Center El Paso, El Paso, USA; 2 Pathology, Texas Tech University Health Sciences Center El Paso, El Paso, USA; 3 Hematology and Oncology, Texas Tech University Health Sciences Center El Paso, El Paso, USA

**Keywords:** sars-cov-2 infection, severe acute respiratory syndrome coronavirus-2, vaccine-induced prothrombotic immune thrombocytopenia (vipit), vaccine-induced thrombotic thrombocytopenia (vitt), amegakaryocytopenia, heparin induced thrombocytopenia (hit), thrombosis with thrombocytopenia syndrome (tts), covid 19

## Abstract

As a global community, we have learned that the manifestations of severe acute respiratory syndrome coronavirus 2 (SAR-CoV-2), infection, or coronavirus disease 2019 (COVID-19), extends far beyond respiratory compromise. Thrombocytopenia is thought to occur secondary to increased platelet consumption. Platelet activation and platelet-mediated immune inflammation contribute towards the thromboembolic complications seen in COVID-19 patients. In this report, the authors present the unusual case of a 75-year-old female with a history of COVID-19 infection who presented with a transient ischemic attack, thrombocytopenia, and amegakaryocytopenia.

## Introduction

The severe acute respiratory syndrome coronavirus 2 (SAR-CoV-2), also known as coronavirus 2019 (COVID-19), is a novel positive-sense single-stranded RNA that took the world by surprise with its multifarious presentations. To date, we are still learning about the extent to which this viral infection affects the human body. Despite our familiarity with certain signs and symptoms of COVID-19 infection, less commonly reported manifestations are now being brought to our attention. Thrombocytopenia, for example, is thought to occur secondary to increased platelet consumption [1]. Platelet activation and platelet-mediated immune inflammation contribute towards the thromboembolic complications seen in COVID-19 patients [1].

As soon as the World Health Organization (WHO) declared COVID-19 infection a pandemic, vaccine manufacturing began with unprecedented speed [2]. Vaccines are pivotal in preventing the transmission of infectious diseases and have a significant role in reducing morbidity and mortality [2]. Four major vaccines were developed and approved on the basis of randomized, blinded, controlled trials: two messenger RNA-based vaccines that include BNT162b2 (Pfizer-BioNTech) and mRNA-1273 (Moderna), and two adenoviral-vectored vaccines that include ChAdOx1 nCov-19 (AstraZeneca), and Ad26.COV2.S (Johnson & Johnson/Janssen, J&J) [1]. A rare and recently discovered syndrome called 'thrombosis with thrombocytopenia syndrome' (TTS), also known as 'vaccine-induced immune thrombotic thrombocytopenia' (VITT) or 'vaccine-induced prothrombotic immune thrombocytopenia' (VIPIT) has been reported in recipients of adenoviral-vectored COVID-19 vaccines like AstraZeneca and the J&J [1-5].

The Centers for Disease Control and Prevention (CDC) has reported approximately four cases of TTS per one million doses of J&J administered [3]. The Vaccine Adverse Event Reporting System (VAERS) surveillance system is a passive surveillance (spontaneous reporting) system for adverse events after immunization “that is jointly administered by CDC and the United States (US) Food and Drug Administration (FDA)” [4,6]. The VAERS database was interrogated for potential cases of TTS received from December 2020 through September 2021. There were 54 incidences of TTS among 14 million users of J&J yielding an incidence of 3.8 per million (about one in 263,000) [4]. Similarly, in a US.-based case series that took place from December 2020 through August 2021, the reporting rates for TTS were 3.83 per million vaccine doses of J&J and 0.00855 per million vaccine doses of the mRNA-based COVID-19 vaccines [6].

A thorough literature review highlights the rarity of TTS and its propensity for fatal outcomes among recipients of J&J and, less commonly, AstraZeneca. Here, the authors describe the rare case of a 75-year-old female with no prior history of vaccination, but rather a recent history of COVID-19 infection that subsequently led to a presentation closely resembling that of TTS.

## Case presentation

A 75-year-old female with a past medical history of well-controlled essential hypertension, COVID-19 pneumonia three months ago, and thrombocytopenia of unknown etiology diagnosed following COVID-19 infection, presented for an episode of transient aphasia lasting eight minutes. She denied having received any immunization prior to or after being diagnosed with COVID-19. The patient was subsequently worked up for a transient ischemic attack (TIA) and thrombocytopenia, concurrently.

A physical examination was notable for scattered petechiae and no neurologic deficits. Laboratory workup revealed a white blood cell (WBC) count of 13.89 × 10^9^/L, red blood cell (RBC) of 4.18 × 10^6^/L, and platelets at 18 × 10^9^/L. An abdominal ultrasound did not show cirrhosis or splenomegaly. There were no signs of infection or other apparent causes of thrombocytopenia, including recent use of any heparin-containing products. The patient was transfused with platelets and started on dexamethasone 40 mg daily and intravenous immunoglobulin (IVIG) 1 g/kg for presumed immune thrombocytopenic purpura (ITP). However, there was no improvement even after one week of therapy. Dexamethasone and IVIG were discontinued and rituximab was administered, instead.

**Figure 1:**
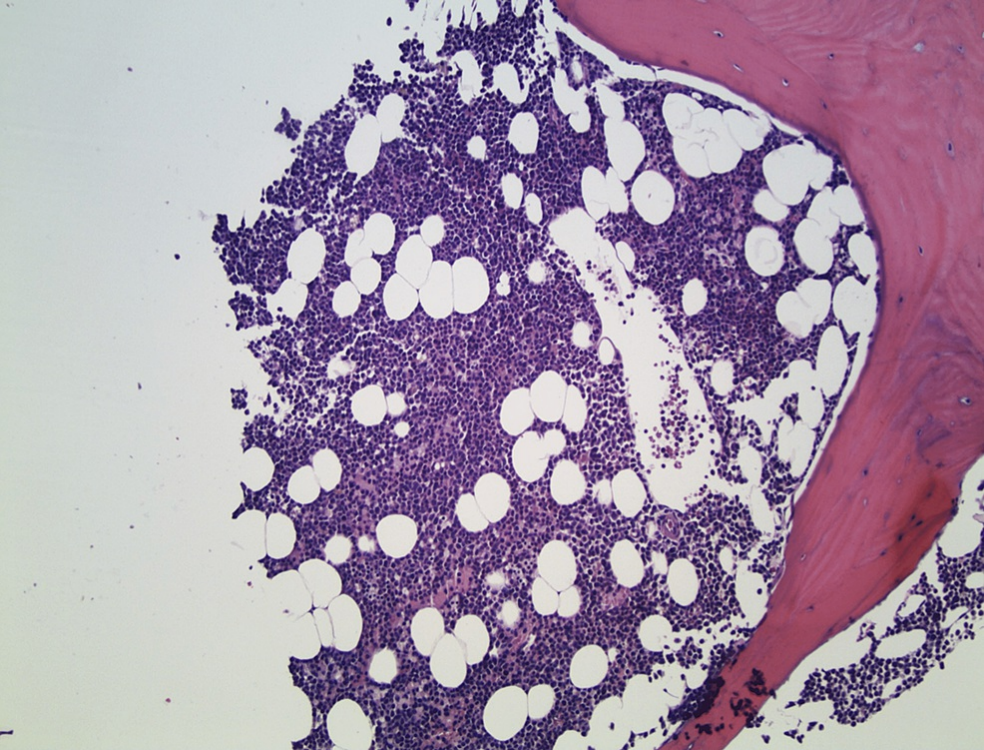
Bone Marrow Biopsy (100X magnification) A section of the bone marrow needle-core biopsy shows a marrow that is hypercellular for age (H&E stain, 100X original magnification). H&E: hematoxylin and eosin

**Figure 2:**
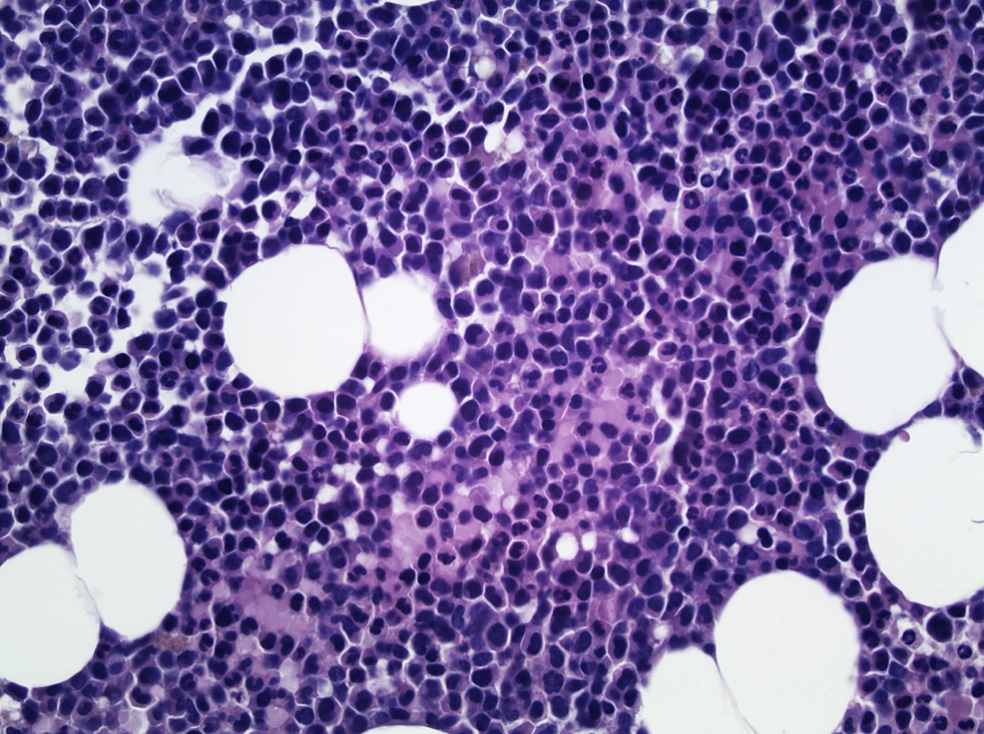
Bone Marrow Biopsy (400X magnification) A section of the bone marrow needle-core biopsy shows hypercellular marrow with increase in mature monocytes (H&E stain, 400X original magnification). H&E: hematoxylin and eosin

A bone marrow biopsy and aspirate were then performed, which showed hypercellular marrow, 70% cellularity, increased granulocytes, decreased erythropoiesis, and absent megakaryocytes (Figures 1, 2). Eltrombopag, a thrombopoietin (TPO) receptor agonist, was then prescribed resulting in a good response with normalization of platelet count after two weeks of therapy.

## Discussion

There are certain triggers other than heparin that can provoke a prothrombotic disorder that strongly resembles heparin-induced thrombocytopenia (HIT) [1,6,7]. Strong clinical similarities exist between TTS and HIT, including thrombocytopenia, thrombosis, coagulation abnormalities or disseminated intravascular coagulation (DIC), and bleeding. Patients with TTS, for example, manifested with cases of thrombosis in some unusual sites, such as cerebral venous sinus thrombosis, which was the most common, splanchnic vein thrombosis, as well as arterial thrombosis [1,6,7]. Interestingly, these patients even shared serologic findings similar to that of HIT [1,5-7]. Laboratory findings revealed concomitant thrombocytopenia, and often the presence of anti-platelet factor 4 (PF4) antibodies. In a study involving 23 patients referred to a specialist hematologist for the evaluation of thrombosis and thrombocytopenia or isolated thrombocytopenia and an elevated D-dimer level following administration of the first dose of the AstraZeneca vaccine, 22 patients tested positive for antibodies to PF4 with one equivocal result [5].

So far, we have discussed TTS as a consequence of vaccination history. An isolated COVID-19 infection without prior vaccination history, however, is seldom reported to lead to a thrombotic and thrombocytopenic presentation. Viral cytopathic effects, multinucleated giant cells, hemophagocytosis, and megakaryocyte recruitment were noted in the autopsies of individuals who died following COVID-19 [8-10]. Significant derangements in the coagulation cascade have been observed in critically ill COVID-19 patients, including elevated D-dimer, fibrinogen, and von Willebrand factor levels [11]. Insult to the lung microvasculature seen in acute respiratory distress syndrome (ARDS) secondary to COVID-19 instigates megakaryocyte fragmentation that may also be a reason for thrombocytopenia [9].

Although the potential for COVID-19 to induce immune thrombocytopenia is appreciated, our patient’s thrombocytopenia was even reflected in the bone marrow. COVID-19 leading to megakaryocyte deficiency has not been reported thus far based on our extensive literature review. Megakaryocytes play an important role in the pathogenic process of COVID-19 through robust gene expression and functional changes in platelets [8,11]. Despite an increased number of activated megakaryocytes, it is thought that this event causes greater platelet release and subsequent activation of the thrombotic pathway [8,9,11,12]. The unique bone marrow biopsy and aspirate from our patient demonstrated a hypercellular marrow, 70% cellularity, increased granulocytes, decreased erythropoiesis, and absent megakaryocytes. It is unclear whether megakaryocyte recruitment or COVID-19-induced megakaryocyte deficiency via an unknown mechanism led to thrombocytopenia.

Consequently, the patient was treated as a TTS-like case with a unique finding of amegakaryocytopenia. The management of this complication appears to mimic HIT and is treated with non-heparin anticoagulants if the platelet count is greater than 50 × 10^9^/L [1,5,13]. High-dose glucocorticoid and IVIG therapy are thought to help increase the platelet count within days and may limit the risk of hemorrhagic transformation, especially when anticoagulation is instituted [5,7]. Plasmapheresis could also temporarily help reduce levels of pathologic PF4 antibodies and partly correct coagulopathy [5]. Other treatment options include rituximab or eltrombopag, which was later given to our patient. The reasoning behind the patient's clinical improvement with eltrombopag remains a mystery and could be attributed to a thrombopoietin deficiency as opposed to a TTS-like picture. Nevertheless, these treatment options have not been adequately studied in patients with a history of COVID-19 infection and a presentation resembling TTS [13]. 

## Conclusions

The risk of COVID-19 remains a serious public health consideration worldwide, and vaccination against SARS-CoV-2 provides critical protection. TTS, or VITT/VIPIT, is an extremely rare but serious adverse event associated with AstraZeneca and J&J. However, it is imperative to recognize that a clinical presentation mimicking TTS can be seen in patients following COVID-19 infection without prior vaccination. The authors report the case of an unvaccinated patient diagnosed with TTS three months following COVID-19 pneumonia and a unique bone marrow biopsy finding. She had a poor response to steroids, IVIg, and rituximab, but responded well to a TPO receptor agonist. The authors recommend that an international blueprint be devised for diagnosing this novel prothrombotic and thrombocytopenic disorder. A standardized management strategy for such an unusual case is also necessary.
